# The TGF-β superfamily as potential therapeutic targets in pancreatic cancer

**DOI:** 10.3389/fonc.2024.1362247

**Published:** 2024-03-04

**Authors:** Rachel R. Tindall, Jennifer M. Bailey-Lundberg, Yanna Cao, Tien C. Ko

**Affiliations:** ^1^ McGovern Medical School, Department of Surgery, The University of Texas Health Science Center at Houston, Houston, TX, United States; ^2^ McGovern Medical School, Department of Anesthesiology, Critical Care, and Pain Medicine, The University of Texas Health Science Center at Houston, Houston, TX, United States

**Keywords:** TGF-β, acute pancreatitis, chronic pancreatitis, pancreatic ductal adenocarcinoma, pancreatic stellate cells

## Abstract

The transforming growth factor (TGF)-β superfamily has important physiologic roles and is dysregulated in many pathologic processes, including pancreatic cancer. Pancreatic cancer is one of the most lethal cancer diagnoses, and current therapies are largely ineffective due to tumor resistance and late-stage diagnosis with poor prognosis. Recent efforts are focused on the potential of immunotherapies in improving therapeutic results for patients with pancreatic cancer, among which TGF-β has been identified as a promising target. This review focuses on the role of TGF-β in the diseased pancreas and pancreatic cancer. It also aims to summarize the current status of therapies targeting the TGF-β superfamily and postulate potential future directions in targeting the TGF-β signaling pathways.

## 1 Introduction

Cytokines mediate the body’s natural response to injury at a systemic level (1). These cytokines can be subcategorized into the transforming growth factor (TGF)-β superfamily, interleukins, interferons, chemokines, and the tumor necrosis factor (TNF) superfamily (2–5). The TGF-β superfamily, one of the major groups, was first described as a family of growth factors released by fibroblasts that stimulated cell growth (6). In 1981, further investigation into these growth factors led to the purification of TGF-β, the first named member of the TGF-β superfamily (7, 8). While studying the purification techniques of this protein, isoforms of TGF-β were discovered, which were called TGF-β1, TGF-β2, and TGF-β3 (9–11). The role of these proteins was further elucidated with the discovery of other members, including the bone morphogenic proteins (BMP)s (12). Currently, 33 proteins are recognized in this superfamily, with subtypes including TGF-βs, BMPs, growth differentiation factors (GDF)s, inhibins, and activins (**Table 1**) (13–16). This mini-review focuses on the role of the TGF-β superfamily in pancreatic diseases, including pancreatic ductal adenocarcinoma (PDAC), and the current therapeutics targeting these pathways.

**Table 1:** Members of the TGF-β superfamily.

Subfamily	Ligand name	Associated gene	Other name	Receptors		R-Smad
				Type I	Type 2	
Transforming Growth Factor (TGF)-β	TGF-β1	*TGFB1*	* *	TGFBRI, ALK-5	TGFBRII	Smad2/3
	TGF-β2	*TGFB2*	* *	TGFBRI, ALK-5	TGFBRII	Smad2/3
	TGF-β3	*TGFB3*	* *	TGFBRI, ALK-5	TGFBRII	Smad2/3
Bone Morphogenic Protein (BMP)	BMP-2	*BMP2*	* *	BMPRIA, BMPRIB	BMPRII, ActRII, ActRIIB	Smad1/5/8
	BMP-3	*BMP3*	* *	ALK-4	BMPRII, ActRII, ActRIIB	Smad2/3
	BMP-4	*BMP4*	* *	BMPRIA, BMPRIB	BMPRII, ActRII, ActRIIB	Smad1/5/8
	BMP-5	*BMP5*	* *	BMPRIA, BMPRIB, ALK-2	BMPRII, ActRII, ActRIIB	Smad1/5/8
	BMP-6	*BMP6*	Vgr1	BMPRIA, BMPRIB, ALK-2	BMPRII, ActRII, ActRIIB	Smad1/5/8
	BMP-7	*BMP7*	* *	BMPRIA, BMPRIB, ALK-2	BMPRII, ActRII, ActRIIB	Smad1/5/8
	BMP-8A	*BMP8A*	* *	BMPRIA, BMPRIB, ALK-2	BMPRII, ActRII, ActRIIB	Smad1/5/8
	BMP-8B	*BMP8B*	* *	BMPRIA, BMPRIB, ALK-2	BMPRII, ActRII, ActRIIB	Smad1/5/8
	BMP-9	*GDF2*	GDF-2	ALK-1, ALK-2	BMPRII, ActRII, ActRIIB	Smad1/5/8
	BMP-10	*BMP10*	* *	ALK-1, ALK-2	BMPRII, ActRII, ActRIIB	Smad1/5/8
Growth Differentiation Factor (GDF)	GDF-1	*GDF1*	* *	ALK-7	BMPRII, ActRII, ActRIIB	Smad2/3
	GDF-3	*GDF3*	Vgr2	ALK-7	BMPRII, ActRII, ActRIIB	Smad2/3
	GDF-5	*GDF5*	BMP-14	BMPRIB	BMPRII, ActRII, ActRIIB	Smad1/5/8
	GDF-6	*GDF6*	BMP-13	BMPRIB	BMPRII, ActRII, ActRIIB	Smad1/5/8
	GDF-7	*GDF7*	BMP-12	BMPRIB	BMPRII, ActRII, ActRIIB	Smad1/5/8
	GDF-8	*MSTN*	myostatin	none	ActRIIB	Smad2/3
	GDF-9	*GDF9*	* *	ALK-5	BMPRII, ActRII, ActRIIB	Smad2/3
	GDF-9B	*BMP15*	BMP-15	ALK-5	BMPRII, ActRII, ActRIIB	Smad2/3
	GDF-10	*GDF10*	BMP-3B	ALK-4	BMPRII, ActRII, ActRIIB	Smad2/3
	GDF-11	*GDF11*	BMP-11	ALK-4, ALK-5	ActRIIB	Smad2/3
	GDF-15	*GDF15*	MIC-1	unknown	unknown	unknown
Nodal	Nodal	*Nodal*	BMP-16	ALK-7	BMPRII, ActRII, ActRIIB	Smad2/3
Inhibin	Inhibin A	*INHA, INHBA*	* *	none	ActRII, ActRIIB	none
	Inhibin B	*INHA, INHBB*	* *	none	ActRII, ActRIIB	none
Activin	Activin A	*INHBA*	* *	ALK-4, ALK-7	ActRII, ActRIIB	Smad2/3
	Activin B	*INHBB*	* *	ALK-4, ALK-7	ActRII, ActRIIB	Smad2/3
	Activin AB	*INHBA, INHBB*	* *	ALK-4, ALK-7	ActRII, ActRIIB	Smad2/3
Lefty	Lefty A	*LEFTY2*	* *	none	ActRII, ActRIIB	none
	Lefty B	*LEFTY1*	* *	none	ActRII, ActRIIB	none
Anti-Mullerian Hormone	AMH	*AMH*	Mullerian-inhibiting substance	BMPRIA, BMPRIB, ALK-2	AMHRII	Smad1/5/8

Adapted from (13–16).

TGFBRI, Type I TGF-β receptor; TGFBRII, Type II TGF-β receptor; BMPRIA, Type IA BMP receptor; BMPRIB, Type IB BMP receptor; ActRII, Type II Activin receptor; ActRIIB, Type IIB Activin receptor.

### 1.1 Physiologic role of TGF-β superfamily

**Figure 1:**
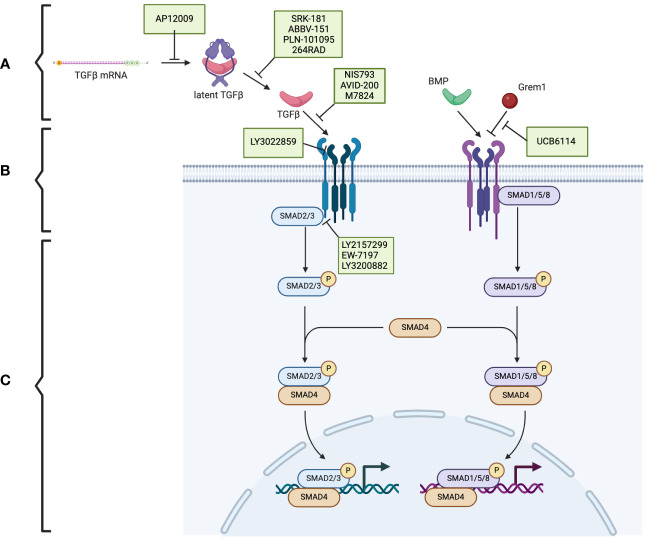
The TGF-β superfamily signaling pathway and the potential therapeutic targets in pancreatic cancer. **(A)** The TGF-β superfamily members. **(B)** TGF-β receptors and the phosphorylation/activation of the intracellular mediators of SMAD2/3 and SMAD1/5/8. **(C)** Downstream of the phosphorylated SMAD2/3 and SMAD1/5/8. Therapeutics (indicated in green boxes) targeting specific steps in the TGF-β superfamily signaling pathway are currently under investigation. Created using BioRender.com.

TGF-β is produced in a latent form. Activation of the latent form is initiated by regulatory T cells with a transmembrane protein, glycoprotein A repetitions predominant (GARP), which binds and cleaves pro-TGF-β to produce latent TGF-β. The latent form is activated by integrins (**Figure 1A**) (17, 18). BMPs, GDFs, and Lefty A and B are produced and processed similarly, with inactive precursors being cleaved and activated by proteases (**Table 1**) (19–21). The activins and inhibins are composed of common subunits and are formed by cleavage of dimerized subunits; inhibins are αβ heterodimers, and activins are ββ homodimers (**Table 1**) (16). The TGF-β superfamily is essential in physiologic functions, including tissue development and differentiation, regulation of immunologic responses, and tissue healing (13). These members activate physiologic activities through canonical and non-canonical signaling (22). Canonical signaling occurs through the SMAD pathway, where receptor-activated (R) SMAD1/5/8 and SMAD2/3 are phosphorylated by receptors following ligand binding (**Figure 1B**). These phosphorylated SMADs then complex with SMAD4 and translocate to the nucleus to regulate the expression of the target genes (**Figure 1C**) (13). Non-canonical pathways can be activated upon the ligand binding to the receptors, such as Erk, involved in epithelial-mesenchymal transition (EMT), and JNK/p38, involved in EMT and apoptosis (23).

These signaling pathways play an essential role in proliferation and in controlling the growth of specific cell types, including epithelial cells, endothelial cells, immune cells, and neuronal cells, through growth inhibition and induction of apoptosis (24). BMPs are specifically involved in developing and maintaining skeletal tissues and are regulated extracellularly by antagonists, including Noggin, Chordin, and Gremlin1 (Grem1) (**Figure 1A**) (14). Additionally, activin A is a critical mesoderm-inducing factor (25), and GDFs are primarily involved in developmental processes (26). Inhibins antagonize activin signaling, and lefty inhibits phosphorylation of SMAD2 and subsequently regulates downstream signaling (**Table 1**) (16, 27).

TGF-β also plays a role in immunoregulation by inhibiting T-lymphocyte proliferation and promoting T-cell differentiation (28–30). Additionally, these proteins play an essential role in fibroblast activation and are involved in routine wound healing; TGF-β1 is secreted by the platelets forming the hemostatic plug and is a chemoattractant for monocytes and fibroblasts essential to tissue repair (31–34).

### 1.2 Pathologic role of TGF-β superfamily

In addition to the essential role of TGF-β in physiologic mechanisms, aberrantly increased TGF-β has been shown to contribute to excess fibrosis (35). Administration of exogenous TGF-β leads to fibrosis in subcutaneous tissues, lung parenchyma, and hepatic tissue (36–38). Furthermore, dysregulation of the TGF-β signaling pathway contributes to carcinogenesis (39). For example, tumor cells have been shown to evade the growth regulation of TGF-β through mutations in the TGF-β receptors and SMAD family (40, 41).

PDAC is currently the third leading cause of cancer-related death (42). Poor outcomes can be attributed to both late diagnosis and a fibrotic tumor microenvironment that surrounds the cancer cells, creating a chemo-resistant barrier. Further understanding of the role of the TGF-β superfamily may elucidate potential targets for novel therapies that could improve patient outcomes.

## 2 Role of TGF-β superfamily in pancreatic disease

TGF-β activity is paradoxical in pancreatic diseases, promoting or suppressing disease progression. The TGF-β superfamily modulates acute pancreatitis (AP) by regulating inflammation and apoptosis through canonical or non-canonical signaling. The TGF-β superfamily also plays distinct roles in the progression of chronic pancreatitis (CP) and PDAC through effects on pancreatic stellate cells (PSCs) and the extracellular matrix (ECM) production.

### 2.1 Acute pancreatitis

AP results from injury to the pancreatic acinar cells, leading to premature activation of pancreatic enzymes and causing pancreatic autodigestion and tissue inflammation (43). Apoptosis and suppression of proliferation have been reported to limit the inflammatory cascade in response to the insult in AP (44). TGF-β is released by various cells at the site of injury and induces apoptosis and suppress the proliferation of pancreatic acinar cells (45). However, this was contradicted by a study showing the induction of apoptosis upon suppressing the TGF-β signaling pathway in pancreatic epithelial cells (46). Additionally, our group has demonstrated that BMP signaling is upregulated in AP and causes dysregulation of autophagic processes. Administration of a BMP antagonist Noggin *in vivo* in a mouse model attenuated AP inflammation, suggesting a proinflammatory role of BMP signaling in AP (47).

In addition to involvement with apoptosis, TGF-β mediates the inflammatory response in AP through T-cell activation. Specifically, TGF-β induces the differentiation of both Th9 and Th17 cells, which are proinflammatory (48, 49). Th17 cells are known to secrete IL-17, associated with increased inflammatory markers and severity of AP (50).

### 2.2 Chronic pancreatitis

CP results from repeated injury to the pancreas from recurrent bouts of AP, which leads to the replacement of normal pancreatic tissue with fibrotic scarring (51). This is primarily facilitated by the activation of PSCs, which secrete growth factors and chemokines such as TGF-β and produce excess ECM (52). The TGF-β secreted by activated PSCs is directly related to the characteristic fibrosis of CP (53). This fibrosis results from increased ECM production by PSCs (54) and inhibition of matrix metalloproteinases, which are involved in ECM degradation (55). Our group has shown that BMPs oppose the fibrogenic function of TGF-β on PSCs in CP by activating the SMAD1/5/8 pathway, which inhibits SMAD2 (56, 57).

Other modulators of TGF-β superfamily pathways are also involved in CP pathophysiology. Our group has shown that Grem1, an endogenous BMP antagonist, is pro-fibrogenic in a CP mouse model (58). Additionally, SMAD7, a known inhibitory SMAD, suppressed TGF-β signaling and modulated CP fibrosis through decreased ECM deposition and decreased inflammatory cell response in an *in vivo* mouse model (59).

### 2.3 Pancreatic ductal adenocarcinoma

The TGF-β superfamily plays dual roles in PDAC, promoting tumorigenesis in some capacities while acting as an inhibitor in others (60). In the early stages of PDAC, TGF-β has been shown to suppress tumor progression by promoting apoptosis and regulation of the cell cycle and promoting the stroma’s development by activating PSCs and increasing stromal production (61). Additionally, BMP2 expression is increased in pancreatic cancer and has variable mitogenic effects on pancreatic cancer cell lines, with a greater capacity to stimulate growth in cell lines with SMAD mutations (62). BMP signaling has also been shown to play a role in EMT through canonical BMP signaling, mediated by Grem1 inhibitory feedback, resulting in a maintenance of heterogeneity (63). Our group has also shown that activated fibroblasts express Grem1 and that increased expression is associated with a more severe tumor stage (64).

## 3 Genetic alterations in PDAC

PDAC is associated with several common mutations in oncogenes and tumor suppressor genes, including mutations in *KRAS, TP53, CDKN2A*, and *SMAD4.* These mutations can affect TGF-β signaling pathways at various points, including the intracellular signaling molecules and receptors (65).

### 3.1 *SMAD4* mutation

*SMAD4* mutations are common in PDAC and are identified in approximately 60% of cases (66). Interestingly, an isolated *SMAD4* mutation does not independently cause cancer; it must be paired with another mutation, such as *KRAS* (67). SMAD4 is part of the intracellular signaling pathway that complexes with activated and phosphorylated SMAD2/3 and SMAD1/5/8 in response to TGF-β and BMP binding their respective receptors (68). Mutation of *SMAD4* results in loss of the tumor suppressor function of canonical TGF-β signaling (69). Loss of *SMAD4* results in decreased T-cell recruitment and a suppressed immune response (70). Additionally, knockout of *Smad4* in a PDAC mouse model has increased tumor sensitivity to host immune control and induced DNA damage (71).

### 3.2 Receptor mutations

Mutations in TGF-β receptors have been identified as disruptions of TGF-β signaling pathways that result in the loss of TGF-β suppressive effects. Studies have shown that mutations in *TGFBR1*, which encodes TGF-β type I receptor (TGFBRI), occur in approximately 1% of cases, and mutations in *TGFBR2*, which encodes TGF-β Type II receptor (TGFBRII), appear in approximately 4% of patients (72). Type III TGF-β receptor mutations also occur and result in increased EMT-associated increased motility and invasiveness (73).

Disruption in the expression of other receptors has also been reported. Deletion of *ACVR1B*, which encodes the ALK-4 receptor for activin A, is associated with a more aggressive cancer phenotype (74). Interestingly, mutations in *BMPRI* are described in patients with hereditary juvenile polyposis. *BMPRI* and *BMPRII* mRNA levels are upregulated in pancreatic cancers, and cells with higher levels have been shown to have more significant metastatic potential (75, 76).

### 3.3 Other mutations

*KRAS* is frequently mutated in human carcinomas and approximately 85% of PDAC cases (77). *KRAS* mutations are often detectable early in disease progression (78). *GREM1* is upregulated in hereditary mixed polyposis syndrome, where duplications of the gene result in increased antagonism of BMP signaling (79). Similar mutations are observed in sporadic intestinal polyps (80). However, mutations in *GREM1* have not been reported in cases of PDAC.

## 4 Therapeutic potential of targeting the TGF-β superfamily

Management options for pancreatic cancer depend primarily on the stage of the cancer when it is diagnosed. Distant metastasis, retroperitoneal invasion, and invasion of the mesenteric root are contraindications to surgical resection. Chemotherapy is the standard of care for metastatic pancreatic cancer. Gemcitabine was considered the first line for a couple of decades following a randomized control trial showing more favorable outcomes than fluorouracil. However, survival for patients treated with gemcitabine was still dismal, with a median survival of 5.65 months (81). This regimen has been improved following the PRODIGE and MPACT studies, which evaluated FOLFIRINOX and albumin-bound paclitaxel plus gemcitabine, showing significant improvement in survival time compared to gemcitabine alone (82, 83). Despite these improved regimens, outcomes remain poor, leading to a focus on the potential of other treatment modalities, including immunotherapy.

Interestingly, chemotherapy has been shown to alter the tumor microenvironment through reprogramming and increased synthesis of chemokines, including TGF-β (84). Thus, TGF-β appears to be involved in the resistance to chemotherapy. Inhibiting TGF-β has become a focus of therapeutic intervention and shows promising results in treating PDAC.

### 4.1 Inhibition of TGF-β signaling

Because of the complexity of the TGF-β signaling pathway, numerous potential targets are under investigation (**Figure 1**). Therapeutic strategies include antisense oligonucleotides, neutralizing antibodies, ligand traps, and small molecule kinase inhibitors. Many of these therapies are being investigated in several cancers, including pancreatic cancer.

#### 4.1.1 Antisense oligonucleotides

Trabedersen (AP12009), specific for *TGFB2* mRNA, reduced *TGFB2* expression in human pancreatic cancer cell lines, resulted in decreased proliferation and migration, and reversed immunosuppressive effects (**Figure 1A**) (85). A phase 2 clinical study showed a good safety profile, with the only identified adverse effect being transient thrombocytopenia and a mean survival of 13.4 months for 61 patients with pancreatic cancer (86). Further clinical trials have yet to be published.

#### 4.1.2 Neutralizing antibodies

In preclinical studies, SRK-181, which specifically targets latent TGF-β1, countered TGF-β-mediated resistance to cancer checkpoint blockade therapy (**Figure 1A**) (87). It is currently under investigation in the DRAGON trial (NCT04291079), a phase 1 clinical trial investigating it as a monotherapy or in combination with anti-PD-L1 in patients with solid tumors, including pancreatic tumors, which has shown no dose-limiting toxicity and adverse effects limited to fatigue, anorexia, and nausea. One patient with pancreatic cancer who was treated with SRK-181 as a monotherapy showed stable disease (88).

Livmoniplimab (ABBV-151) targets GARP-TGF-β1 and prevents the release of active TGF-β1 (**Figure 1A**). It is currently under investigation in a phase 1 trial (NCT03821935), investigating it as a single agent or combined with Budigalimab in patients with locally advanced or metastatic solid tumors. This clinical trial is still in the recruiting phase, and preliminary results are not yet available (89).

PLN-101095 targets integrin αvβ8 and αvβ1 and prevents activation of TGF-β (**Figure 1A**). It has shown enhanced response to standard chemotherapy regimens in preclinical studies (90) and is currently in a phase 1 clinical trial (91). Additionally, 264RAD inhibits integrin αvβ6 and has shown promising results in preclinical trials (92); however, further clinical trials have not been pursued.

NIS793 binds and neutralizes active TGF-β with high affinity and has been shown to decrease fibroblasts and enhance tumor cell chemosensitivity (**Figure 1B**) (93). In a phase 1b trial (NCT02947165), 120 patients, of which ten had pancreatic cancer, were treated with NIS793 as a monotherapy or in combination with spartalizumab. Partial response was observed in 2.5% of patients, and stable disease was observed in 24.2% of patients. While no dose-limiting toxicity was observed, nearly half experienced an adverse event, most commonly rash (94). A phase 2 trial (NCT04390763) and phase 3 trial (NCT04935359) are ongoing to evaluate the drug’s effect in patients with metastatic pancreatic ductal adenocarcinoma.

LY3022859 targets the type II TGF-β receptor and inhibits signaling activation (**Figure 1B**). A phase 1 trial (NCT01646203) was discontinued due to patients developing uncontrollable cytokine release syndrome (95).

#### 4.1.3 Ligand traps

AVID-200 is explicitly designed to resemble the receptor ectodomain for TGF-β1 and TGF-β3 and has been shown to enhance the efficacy of immune checkpoint inhibitors in preclinical trials (**Figure 1B**) (96, 97). It recently underwent a phase 1 clinical trial (NCT03834662) for solid tumors, including PDAC (98).

Activated T-cells present PD-1 on the surface, which can be exploited by tumor cells expressing PD-L1. PD-L1 binding to PD-1 inactivates the T-cells and prevents the T-cell-regulated destruction of the tumor cells (99). Thus, the PD-1 signaling pathway has been identified as a promising target for cancer immunotherapy (100). Specific interest has arisen in dual inhibition of the PD-1 and TGF-β signaling pathways, which is hypothesized to enhance the anti-tumor activity (101). Bintrafusp alfa (M7824) is a bifunctional fusion protein with a type II TGF-β receptor fused to an antibody against PD-L1 (**Figure 1B**) (102), which has undergone a phase 1 clinical trial (NCT02517398) that included five patients with pancreatic cancer. Three patients had a response of stable disease, one of partial response, and one of progressive disease (103).

#### 4.1.4 Small molecule kinase inhibitors

Galunisertib (LY2157299) is an oral drug that inhibits the type I TGF-β receptor kinase and down-regulates the phosphorylation of SMAD2 (**Figure 1B**) (104). In phase 1 and 2 clinical trials, a combination of galunisertib and gemcitabine resulted in an improved survival time of 8.9 months compared to 7.1 months in patients treated with just galunisertib with minimal increase in toxicity in patients with locally advanced or metastatic pancreatic adenocarcinoma (105).

Vactosertib (EW-7197), a type I TGF-β receptor inhibitor, has been shown to augment gemcitabine and decrease the expression of ECM components, improving the sensitivity of pancreatic cancer cells to gemcitabine (**Figure 1B**) (106). It also has synergistic effects when combined with T1-44, an inhibitor of PRMT5 methyltransferase (107). It has been investigated in a phase 1b clinical trial in combination with FOLFOX in sixteen patients with pancreatic ductal adenocarcinoma; three patients had a partial response, and five had stable disease (108).

LY3200882, an oral type I TGF-β receptor inhibitor, was investigated in a phase 1 clinical trial (**Figure 1B**). LY3200882 was used in combination with gemcitabine and nab-paclitaxel in twelve patients with pancreatic cancer. Six of the twelve patients had partial responses, and all but one demonstrated decreased tumor size (109).

### 4.2 Other targets

In addition to TGF-β, other members of the TGF-β superfamily are promising targets for cancer therapeutics. Interestingly, ginisortamab (UCB6114), an antibody that neutralizes Grem1 and blocks its antagonistic effects on BMP signaling, has been shown to restore BMP signaling pathways in human colorectal cancer cell lines and fibroblasts (**Figure 1B**) (110). It is currently being evaluated by a phase 1/2 clinical trial (NCT04393298) in advanced solid tumors, including pancreatic adenocarcinomas.

GDF-15 has also been identified as a potential target. While the exact signaling pathway has yet to be elucidated, recent studies have identified a unique GDF-15 receptor glial cell-derived neurotrophic factor family receptor α-like (GFRAL) and have shown that GDF-15 inhibits leukocyte integrin activation and T cell migration, which is reversed with neutralization of GDF-15 (111, 112). Visugromab (CTL-002), a neutralizing antibody of GDF-15, is currently under evaluation in phase 2a of the GDFATHER trial (NCT04725474) and is showing promising results in combination with nivolumab in advanced non-small cell lung cancer and urothelial cancer.

## 5 Discussion

TGF-β and associated proteins undoubtedly play a role in the development of pancreatic disease and disease progression from AP to CP to PDAC. However, the heterogeneous nature of pancreatic tissue and the dynamic role of the TGF-β superfamily and associated signaling pathways result in nuanced implications for therapeutics that target these pathways.

Systemic therapies such as chemotherapy have been the standard of care for patients with pancreatic cancer following trials such as PRODIGE 4/ACCORD 11 and MPACT; however, outcomes remain very poor with short survival times (83, 113). This has led to an interest in targeted therapies such as immunotherapy, which modulate a patient’s immune system response. Such therapies have survival benefits in several types of solid tumors, including upper gastrointestinal tumors and colorectal cancers (114, 115). However, similar benefits from these immunotherapies have not been seen in pancreatic cancer, mainly because pancreatic tumors are immunologically cold due to the unique immunosuppressive tumor microenvironment with limited immune cells (116). Thus, current strategies seek to target components of the microenvironment that contribute to this immunosuppression to improve the responsiveness of tumors to immunotherapy (117). Subsequently, TGF-β signaling pathways became targets of interest, given the role of TGF-β in immunosuppression and ECM production.

Several therapeutics that target TGF-β and associated pathway molecules are currently under investigation in early clinical trials for solid tumors, including pancreatic tumors. These targets include modulating the TGF-β signaling pathway directly and targeting other proteins in the superfamily, such as Grem1, which inhibits BMP signaling. Additionally, antagonism of BMP has been suggested as a potential target to block and reduce pancreatic cancer invasiveness (118). However, due to the context-dependent manner of BMP signaling, the efficacy of such therapeutics varies greatly, and further investigation into the subtleties of BMP signaling in both oncogenic and tumor-suppressive functions is warranted (119).

Because the TGF-β superfamily has heterogenous roles in pancreatic tumor development, the effectiveness of these therapies has yet to be fully elucidated. TGF-β signaling pathways are involved in immunosuppression and ECM production, but TGF-β pathways also regulate cell cycle progression. Regardless, data from the recent clinical trials suggest hopeful results. Side effect profiles were essentially minimal, but the potential adverse effects of TGF-β targeting drugs when delivered systemically should be a point of investigation in future studies, as blockage of the signaling pathways has previously been shown to have contradictory effects depending on cell type (120). Targeted drug delivery to the pancreatic tumor microenvironment may help mitigate such effects.

Ultimately, definitive management of pancreatic disease will likely require a multifaceted treatment plan due to the heterogeneous nature of the disease processes. TGF-β has been shown to augment the microenvironment, which likely contributes to the characteristic resistance and poor outcomes of PDAC. Inhibition of these signaling pathways shows promising results in boosting the effects of traditional therapeutics. Pairing modulators of TGF-β signaling pathways with conventional systemic treatments such as chemotherapy and other immune modulators such as PD-1 inhibitors will address the mechanisms of resistance that have contributed to the poor outcomes of pancreatic cancer and allow for a more comprehensive treatment regimen.

## Author contributions

RT: Conceptualization, Validation, Writing – original draft, Writing – review & editing, Funding acquisition, Investigation, Visualization. JB-L: Conceptualization, Writing – review & editing, Funding acquisition, Investigation, Visualization. YC: Conceptualization, Supervision, Validation, Writing – review & editing, Investigation, Project administration, Visualization. TK: Conceptualization, Supervision, Writing – review & editing, Funding acquisition, Investigation, Project administration.
